# A novel approach for microRNA in situ hybridization using locked nucleic acid probes

**DOI:** 10.1038/s41598-021-83888-5

**Published:** 2021-02-24

**Authors:** Isabella W. Paulsen, Michael Bzorek, Jesper Olsen, Birgitte Grum-Schwensen, Jesper T. Troelsen, Ole B. Pedersen

**Affiliations:** 1 Department of Clinical Immunology, Zealand University Hospital, Koege, Ringstedgade 77B, 4700 Naestved, Denmark; 2 Department of Science and Environment, Roskilde University, Roskilde, Denmark; 3 Department of Pathology, Zealand University Hospital, Roskilde, Denmark; 4 Department of Surgery, Zealand University Hospital, Koege, Denmark

**Keywords:** Biological techniques, Biotechnology, Cell biology, Molecular biology, Biomarkers, Diseases, Medical research, Molecular medicine, Diagnosis

## Abstract

Identification of target tissue microRNAs (miR) using in situ hybridization (ISH), with digoxigenin-labeled locked nucleic acid (LNA) probes, is influenced by preanalytic parameters. To determine the best retrieval method for common microRNAs, a multiblock composed of paraffin-embedded tonsil, cervix, placenta, and hyperplastic prostate tissue were included. Tissue were fixed in 10% formalin in a range of 5–144 hours (h). Cut sections (5 μm) from the multiblock were subjected to combinations of pretreatment procedures: variable periods of proteinase K (PK) digestion or Heat-induced microRNA Retrieval (HmiRR) using target retrieval solution (TRS) pH 6.1 or 9, with or without enzymatic treatment (pepsin). Results for the overall categories: TRS pH 9 versus PK; *p* = 2.9e−23, TRS pH 9 versus TRS pH 6.1; *p* = 1.1e−14, TRS pH 6.1 versus PK; *p* = 2.9e−03. A long fixation time, resulted in the best microRNA preservation and staining intensity (long vs. short: *p* = 3.5e−47, long vs. moderate: *p* = 1.6e−44, moderate vs. short: *p* = 4.3e−16), was enhanced using HmiRR TRS pH 9 with or without pepsin providing high sensitivity and specificity. These observations conflict with other ISH techniques (e.g., messenger ribonucleic acid), which typically require shorter fixation periods, and therefore, further studies are warranted.

## Introduction

Over the past decade, noncoding RNA (ncRNA), including microRNAs, has become a hot research topic^1–3^. Today, many different molecular methods are used for detection of microRNAs in tissue samples. Microarray and Next-Generation Sequencing (NGS) can be used for first phase validation. Quantitative RT-PCR is a popular tool for second phase validation analysis. For target validation of a given microRNA, tools such as luciferase reporter assays and northern blotting can be used for cell cultures. However, these methods only reveal the microRNA profile of the whole sample^4,5^ and not where or from which cells the detected microRNAs originate. Therefore, to further elucidate the pathogenetic role or function of different microRNAs, it is of great interest to identify the specific location of microRNA expression in tissue. From that perspective, another molecular tool, microRNA in situ hybridization can be used to visualize and locate the site of expression in a formalin-fixed paraffin-embedded (FFPE) tissue sample^6,7^.

In the field of ISH, proteolysis^8,9^ or proteinase K treatment^10,11^ is often recommended for retrieval of specific nucleic acid sequences in FFPE samples. However, the use of proteases can be difficult because the enzyme efficacy varies from batch to batch. Therefore, the concentration, digestion time and/or temperature must be adjusted and optimized each time ISH is performed. Excessive digestion of the tissue results in poor morphology and loss of microRNA. Several studies have shown that the appropriate buffers at high temperatures can also be used to recover DNA/RNA^12,13^, but only few reports support the use of Heat-induced microRNA Retrieval with LNA microRNA probes and ISH^14–16^. Based on our own experience, performing microRNA-ISH using vendor recommended protocol settings is often troublesome (typically based on enzymatic digestion), labor intensive, and in the worst cases inaccurate. Therefore, the need for a ‘standardized’ system that can easily be implemented for both routine and research purposes is highly wanted.

The present study provides a “pretreatment test battery” model inspired by Taylor et al.^17^ for optimization of microRNA retrieval, with the aim of improving microRNA ISH assays of FFPE tissue using specific LNA probes^18^. The pretreatment procedures were chosen based on our own experience for effective retrieval of antigens (immunohistochemistry). In addition, we compared the morphology and signal intensity across tissues after using different pretreatment procedures and variable fixation length/time in 10% buffered formaldehyde (formalin) solution.

## Results

**Figure 1:**
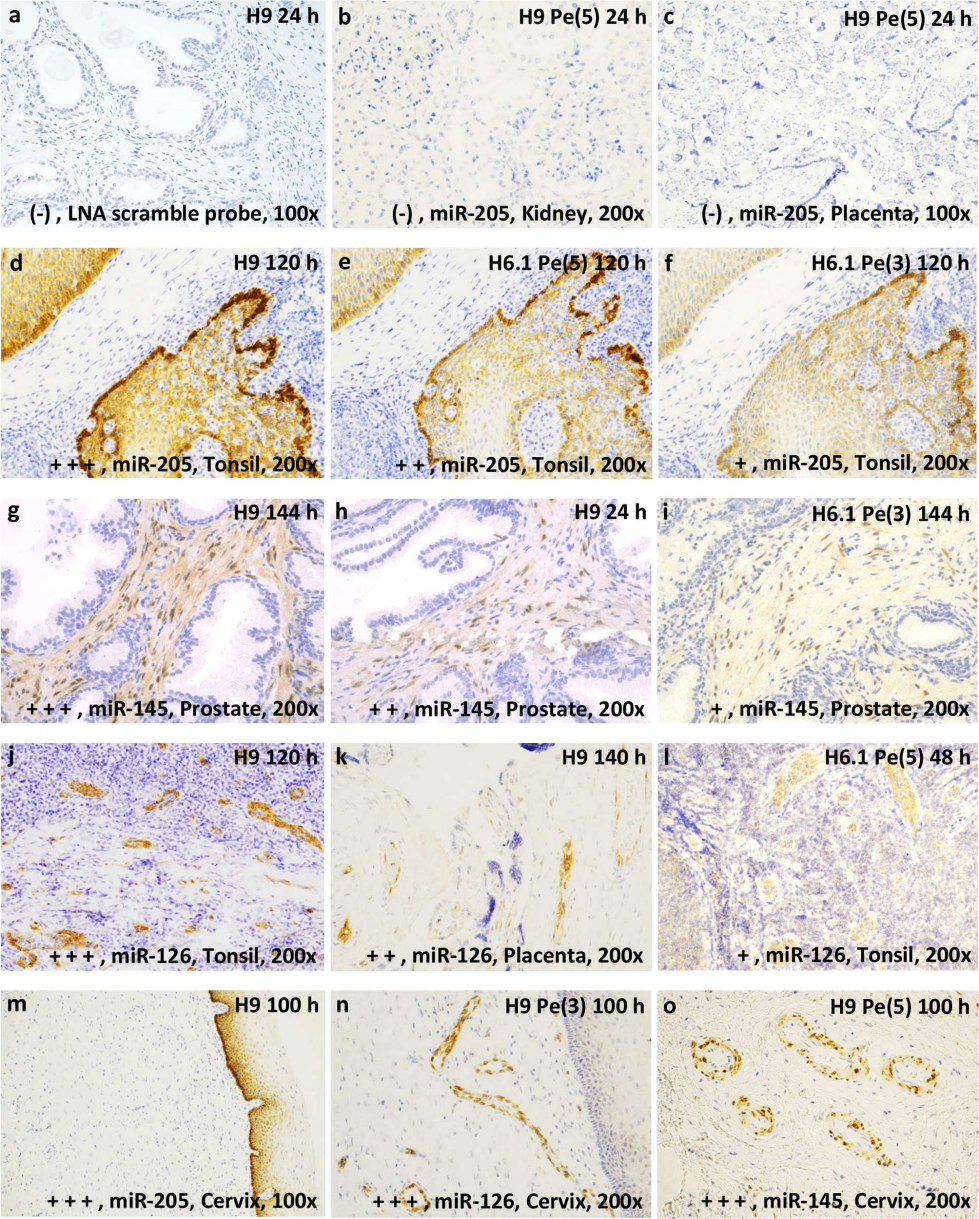
In situ hybridization images of the rating scale. The pictures were taken at a magnification ×200 or ×100 optic zoom with light microscopy. Blue color = nuclear staining (background staining). Brown color = probe staining. Rating is indicated with + (plus), hence, (−) = 0 and + to +  +  + (1 to 3). (**a**) Negative control, locked nucleic acid (LNA) scramble probe in prostate tissue. (**b**, **c**) absent probe staining of microRNA miR-205-5p (miR-205) in placenta- and kidney tissue. (**d**–**f**) staining of miR-205 in tonsil tissue. (**g**–**i**) staining of miR-145-5p (miR-145) in prostate tissue. (**j**–**l**) staining of miR-126-3p (miR-126) in tonsil or placenta tissue. (**m**–**o**) No cross-hybridization of the probes miR-205, miR-126 and miR-145 in cervical tissue: (**m**) miR-205 expression in basal epithelia cells, (**n**) miR-126 in endothelia cells, (**o**) miR-145 smooth muscle cells around endothelia cells. Treatment: Heat-induced microRNA Retrieval (HmiRR) using target retrieval solution pH 6.1 or 9 (H6.1 or H9). Enzymatic exposure: pepsin, Pe. Exposure time for proteolysis treatment is given in minutes and presented in (). Period of formalin fixation is given in hours, h. (**a**) H9 24 h, (**b**) H9 Pe(5) 24 h, (**c**) H9 Pe(5) 140 h, (**d**) H9 120 h, (**e**) H6.1 Pe(5) 120 h, (**f**) H6.1 Pe(3) 120 h, (**g**) H9 144 h, (**h**) H9 24 h, (**i**) H6.1 Pe(3) 144 h, (**j**) H9 120 h, (**k**) H9 140 h, (**l**) H6.1 Pe(5) 48 h, (**m**) H9 100 h, (**n**) H9 Pe(3) 100 h, (**o**) H9 Pe(5) 100 h.

The first part of the protocol involves pretreatment, denaturation and hybridization. The second part of the protocol involves detection and visualization of microRNA expression in tissue. A paraffin multiblock with formalin-fixed tissue cores was prepared (Supplementary Figure S1). Kidney, placenta, prostate, tonsil and uterine cervical tissue were selected based on the known expression patterns of three microRNAs: miR-205-5p (squamous epithelium), miR-145-5p (smooth muscle cells) and miR-126-3p (endothelial cells)^19,20^. A LNA scramble probe was included as negative control. No background staining was detected with LNA scramble probes and all probes revealed expected reaction patterns. The individual tissue cores in the multiblock sections serve as positive and negative controls for the respective LNA microRNA probes applied in this study, confirming the specificity of the staining protocol (Fig. 1).

Pretreatments were divided into proteolytic (Proteinase K and pepsin addition) and HmiRR with low or high pH buffer (TRS pH 6.1/TRS pH 9). For fixation of clinical samples, 1–3 days of fixation has been proposed^20^, and 16–32 h has been suggested as the optimal fixation condition for obtaining high-quality RNA^21^. To address optimal fixation conditions for microRNA ISH, tissues were fixed for different time intervals: short (5–24 h), moderate (30–76 h) and long (100–144 h).

Supplementary Tables S1–S3 display the mean rating of all pretreatments stratified into different fixation and tissue type groups. The mean ratings cover the given experimental combinations for the three LNA microRNA probes. As examples of the rating scale: negative (0) and 1 to 3, see Fig. 1.

**Figure 2:**
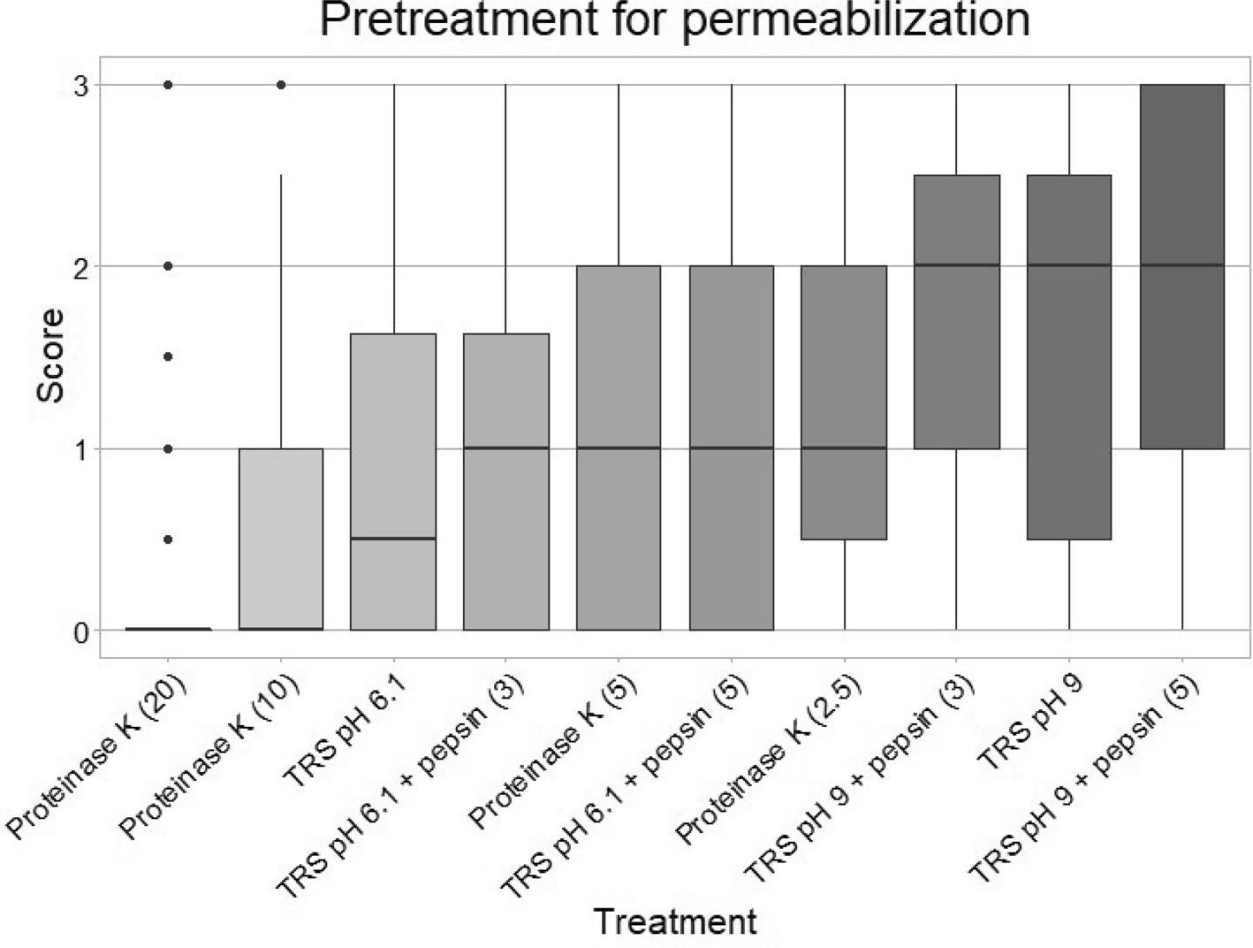
Boxplot of score distribution according to pretreatment of formalin-fixed paraffin-embedded tissues. Choice of pretreatment for permeabilization: Heat-induced microRNA Retrieval in Target Retrieval Solution (TRS) pH 6.1, TRS pH 9 or proteolysis with proteinase K. Exposure time for proteolysis treatment is given in minutes and presented in (). All scores are given regardless of tissue type, hence revealing the overall score of probe-staining effectivity. The x-axis shows type of treatment. The y-axis shows the mean score of the probe staining on a scale from 0 to 3, where 0 = no staining of the probe, and 3 is strong staining of the probe. The darker the color, the better the staining. The figure was constructed with the statistical software R.

The numeric rating data are aggregated, and the different scores are stratified according to tissue and fixation time to compare the different pretreatments (Table 1). The top four best pretreatments according to mean intensity were as follows: (1) TRS pH 9 treatment with pepsin for 5 min (mean = 1.8), (2) TRS pH 9 without additional pepsin (mean = 1.7), (3) TRS pH 9 with pepsin for 3 min (mean = 1.7), (4) proteinase K treatment for 2.5 min (mean = 1.4) (Fig. 2 and Table 1). These top four pretreatments were not statistically significantly different (Supplementary Table S4).

**Table 1:** MicroRNA in situ hybridization, ISH, result table.

Fixation time (h)	Time (min)	Cervix			Prostate			Tonsil			Placenta			Statistics				
		5	30	100	24	72	144	6	48	120	6	36	140	Mean 1	SD 1	ICC 1	*p* value 1	Global
Proteinase K	2.5	0.6	1.4	1.9	1.4	1.7	2.5	0.7	1.7	2.2	0.3	0.7	1.4	1.4	0.9	0.9	8.0e−03	0.9 0.9 2.9e−23
Proteinase K	5	0.3	0.9	1.7	1.1	1.5	2.2	0.4	1.3	2.3	0.0	0.4	1.7	1.1	0.8	0.9	6.0e−05	
Proteinase K	10	0.2	0.4	1.3	0.8	1.0	1.6	0.2	0.3	1.3	0.0	0.4	0.9	0.7	1.0	0.9	1.0e−10	
Proteinase K	20	0.1	0.0	0.3	0.5	0.5	1.2	0.0	0.1	0.3	0.0	0.0	0.0	0.2	0.3	0.9	1.1e−20	
TRS pH 6.1	20	03	0.6	1.7	0.6	0.9	1.8	0.4	0.9	1.8	0.1	0.2	0.9	0.9	0.4	0.9	1.3e−08	1.1 0.8 1.1e−14
TRS pH 6.1 + pepsin	20 + 3	0.4	0.8	1.7	0.7	1.3	1.9	0.7	1.2	2.1	0.0	0.2	1.4	1.0	0.2	0.9	2.1e−06	
TRS pH 6.1 + pepsin	20 + 5	0.8	0.8	2.3	1.0	1.4	2.3	0.6	1.1	2.1	0.2	0.2	1.6	1.2	0.3	0.8	5.0e−04	
TRS pH 9	20	1.2	1.4	2.8	1.5	2.0	2.8	1.3	1.8	2.7	0.3	0.5	2.0	1.7	0.3	0.9	5.6e−01	1.8 0.9 –
TRS pH 9 + pepsin	20 + 3	1.4	1.3	2.8	1.4	1.8	2.6	1.3	1.8	2.6	0.3	0.7	2.3	1.7	0.3	0.9	4.2e−01	
TRS pH 9 + pepsin	20 + 5	1.4	1.6	2.9	1.4	1.9	2.6	1.3	2.2	2.9	0.3	0.8	2.5	1.8	0.1	0.8	–	
Mean 2		0.7	0.9	2.0	1.0	1.4	2.2	0.7	1.2	2.0	0.1	0.4	1.5					
SD 2		0.6	0.7	0.9	0.7	0.8	0.9	0.5	0.8	0.9	0.2	0.5	0.8					
ICC 2		0.9	0.8	0.7	0.9	0.8	0.8	0.9	0.9	0.7	0.9	0.7	0.2					
*p* value 2		7.9e−14	6.1 e−14	2.5 e−02	4.7 e−14	1.0 e−13	–	1.5 e−14	2.1 e−10	2.4 e−01	7.4 e−17	2.2 e−16	9.7 e−11					

The means of all three ISH experiments with the miR-205-5p, miR-145-5p and miR-126-3p probes, due to the given parameters, are presented within the largest square (main table). The upper horizontal bar shows the included tissue, and the numbers indicates the period of formalin fixation in hours. The first column from the left indicates pretreatment for permeabilization: proteinase K or Heat-induced microRNA Retrieval (target retrieval solution (TRS) pH 6.1 or TRS pH 9) with (w/) or without (w/o) pepsin addition. The second column shows exposure time for the given treatment. The additional number after the (+) presents enzymatic treatment w/ pepsin in minutes (min). The five columns to the right and four rows below the main table display statistics for the wide and longitudinal data: each test according to the ten pretreatments, regardless of tissue type and fixation period or the twelfth tissue-fixation categories. Mean 1 and 2 presents the mean across data points for the given pretreatment or tissue-fixation category SD 1 and 2, standard deviation. ICC 1 and 2, Intraclass correlation coefficients. *p* value 1 and 2, pairwise Wilcoxon signed-rank test relative to the best rated, assigned as (−). Global, the statistics are gathered into three categories: (1) Proteolysis; proteinase K (2.5–20 min), (2) TRS pH 6.1 (w/ and w/o pepsin) and (3) TRS pH 9 (w/ and w/o pepsin). Thus, the first number is the mean. The second is standard deviations. The third is *p* value of pairwise Wilcoxon signed-rank test relative to the best rated category, assigned as (−).

Comparing all the treatments with the best rated treatment (TRS pH 9 with pepsin for 5 min) revealed statistically significant differences from those with the lowest intensity including TRS pH 6.1 with pepsin for 5 min (*p* = 5.0e−04), proteinase K for 20 min (*p* = 1.1e−20), proteinase K for 10 min (*p* = 1.0e−10), proteinase K for 5 min (*p* = 6.0e−05), TRS pH 6.1 (*p* = 1.3e−08) and TRS pH 6.1 with pepsin for 3 min (*p* = 2.1e−06) (Table 1).

**Figure 3:**
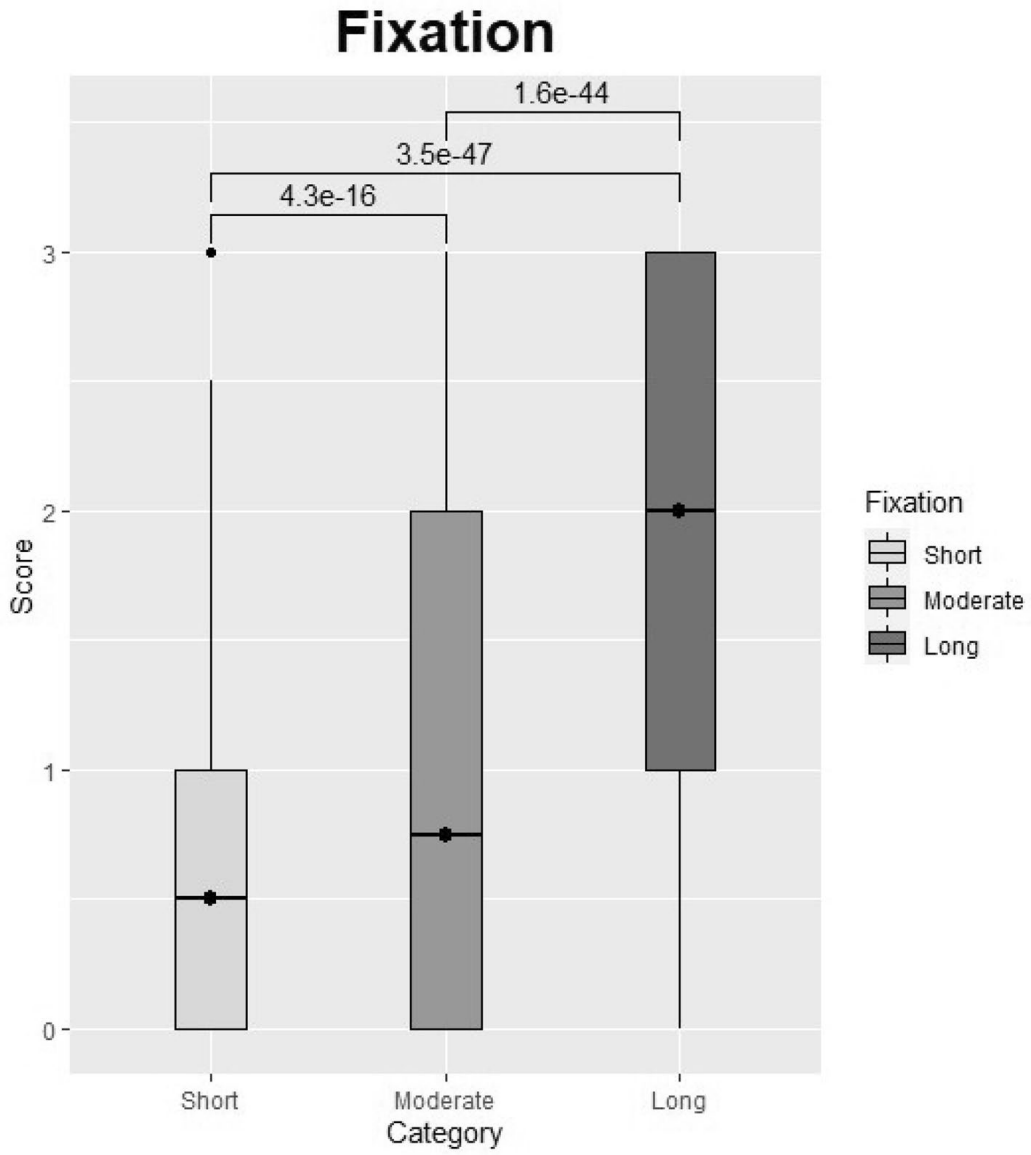
Overall fixation strategies regardless of treatment. Categories: Short = tissue fixation for 5–24 h, Moderate = tissue fixation for 30–76 h, Long = tissue fixation for 100–144 h. All horizontal bars indicate statistical comparison of groups. All numbers above the horizontal bars are *p* values determined using a Wilcoxon signed-rank test. The figure was constructed with the statistical software R.

**Figure 4:**
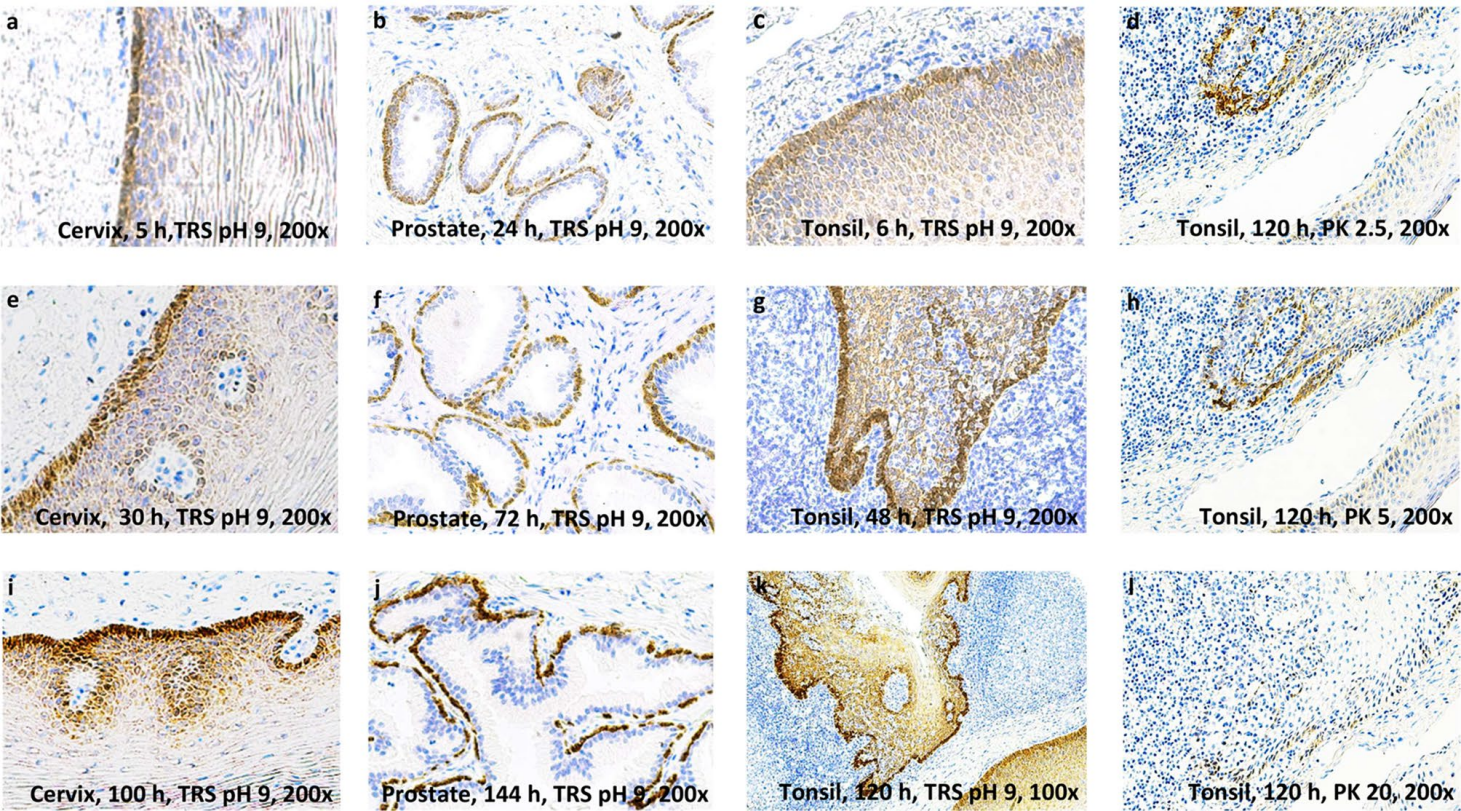
In situ hybridization. The images were taken at ×200 or ×100 (**k**) optic zoom with light microscopy. All tissues display miR-205-5p staining (brown staining). MiR-205-5p expression is restricted to basal epithelial cells. (**a**–**c**, **e**–**g**, **i**–**k**) display Heat-induced microRNA Retrieval using Target Retrieval Solution (TRS) pH 9 (20 min at 100 °C) on prostate, tonsil and cervical tissue fixed in formalin for variable lengths of time (hours, h). Proteinase K (PK) digestion on tonsil tissue: (**d**) (2.5 min), (**h**) (5 min) and (**l**) (20 min). (**a**, **e**, **i**) display miR-205-5p staining in cervical tissue fixed in formalin for 5–100 h. (**b**, **f**, **j**) display miR-205-5p staining in prostate tissue fixed for 24–144 h. (**c**–**d**, **g**–**h**, **k**, **l**) display miR-205-5p staining in tonsil tissue. (**a**, **b**, **c**) display tissues fixed for 5–24 h (Short fixation time). (**e**, **f**, **g**) shows tissues fixed for 30–72 h (moderate fixation time). (**d**, **h**–**l**) shows tissues fixed for 100–144 h (long fixation time).

By comparing different fixation times for the different tissues and categorizing fixation time into three groups (5–24 h, 30–72 h, and 100–144 h), we found that longer fixation periods led to higher signal intensity and better morphology across treatments. Thus, fixing tissues for 5–24 h, 30–72 h or 100–144 h resulted in mean intensity values of 0.64, 0.99, and 1.91, respectively (Table 2), across tissues and choices of treatment. Hence, long fixation (100–144 h) seems most efficient for microRNA preservation in tissue (Figs. 3, 4). Statistically significant differences were found among the following fixation categories: short versus long (*p* = 3.5e−47), short versus moderate (*p* = 4.3e−16) and moderate versus long (*p* = 1.6–44) (Table 2, Fig. 3). Figure 4 demonstrates how extension of the period for fixation enhanced probe staining intensity in tissues treated with HmiRR using TRS pH 9 (20 min in 100 °C). In contrary, extension of the period for proteinase K treatment resulted in digestion of the tissue and as a result in poor probe staining intensity.

**Table 2:** Overall result table.

Fixation	Category	Mean	*p* values	Short	Moderate
5–24 h	Short	0.64	Moderate	4.3e−16	–
30–76 h	Moderate	0.99			
100–144 h	Long	1.91	Long	3.5e−47	1.6e−44

Permabilization	Category	Mean	*p* values	Proteolysis	TRS pH 6.1
Proteolysis	Proteinase K	0.9	TRS pH 6.1	2.9e−03	–
TRS pH 6.1	w/w/o pepsin	1.1			
TRS pH 9	w/w/o pepsin	1.8	TRS pH 9	2.9e−23	1.1e−14

The first column to the left presents the fixation time given in hour intervals and permeabilization categories: Proteolysis, Target Retrieval Solution (TRS) pH 6.1 and TRS pH 9. Second column, category of hour interval: short (5–24 h), moderate (30–76 h), long (100–144 h) and category of permeabilization: proteinase K or with (w/) or without (w/o) pepsin. Third column, Mean: the mean of the scores given in each category (Category). The fourth to the sixth column: a pairwise comparison of all categories using a Wilcoxon rank sum test. The fourth column and upper bar of column six and five displays the category to be compared (short, moderate or long). The numbers in column five and six display the *p* value of the given comparison of the categories. The *p* values were adjusted using the Benjamini and Hochberg procedure.

**Figure 5:**
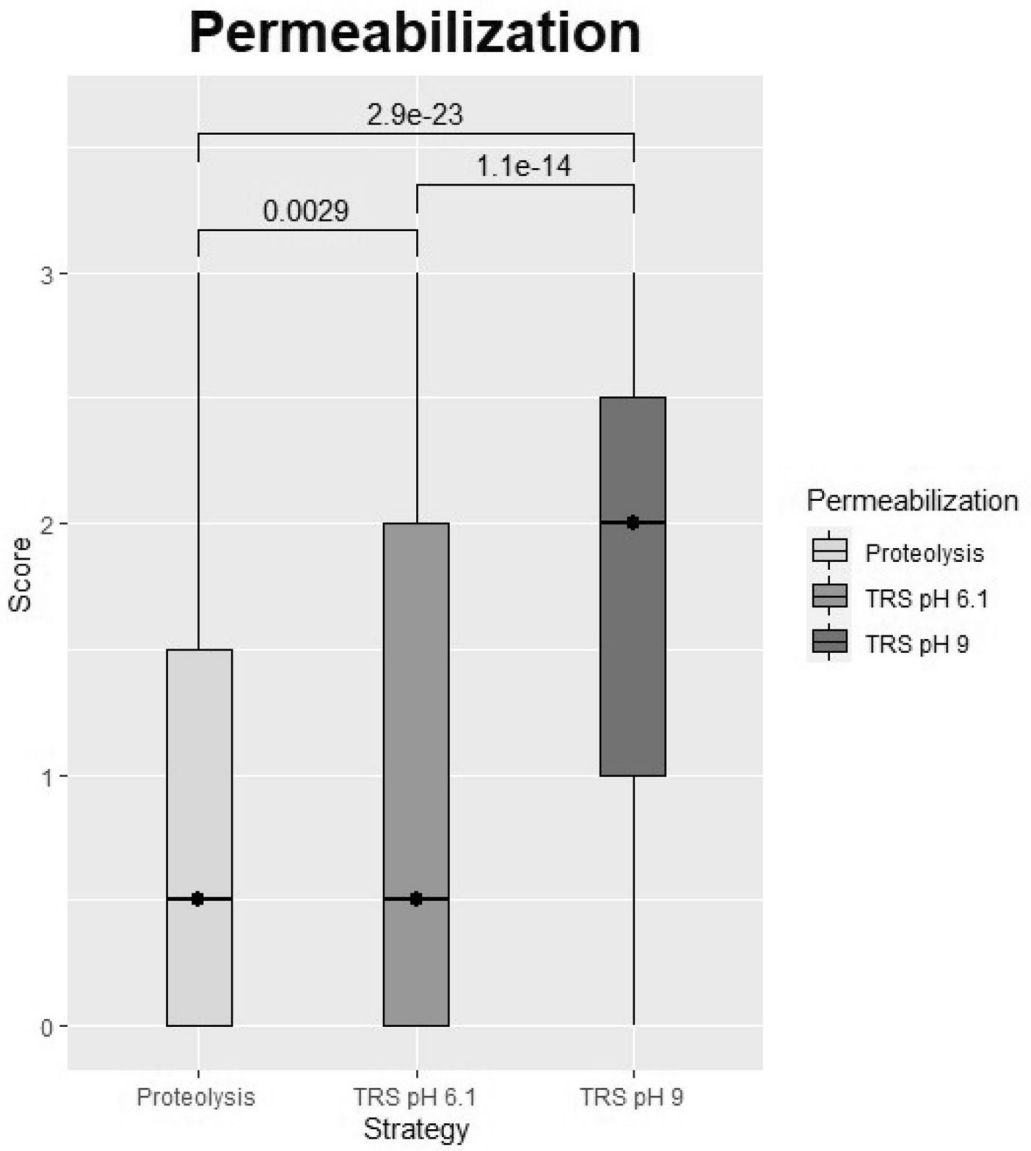
Overall permeabilization strategies. Proteolysis covers all treatment with proteinase K, regardless of the duration of exposure (2.5–20 min). TRS pH 6.1 = Heat-induced microRNA Retrieval (HmiRR) with Target Retrieval Solution (TRS) pH 6.1 regardless of enzymatic treatment with pepsin. TRS pH 9 = HmiRR with TRS pH 9 regardless of enzymatic treatment with pepsin. All horizontal bars indicate statistical comparison of groups. The numbers above the horizontal bars are *p* values determined using a Wilcoxon signed-rank test. The figure was constructed with the statistical software R.

When looking at time of fixation and type of treatment combined dividing them into three categories, namely: proteinase K (regardless of exposure time), HmiRR using TRS pH 6.1 (with or without pepsin) and HmiRR with TRS pH 9 (with or without pepsin) (Table 1), the highest score was obtained using HmiRR with TRS pH 9 (Global mean = 1.8), and thus, this seems to be the best treatment choice for permeabilization of FFPE tissues regardless of fixation time (Fig. 5). HmiRR with TRS pH 9 preserved morphology and had better intention was statistically significantly better than TRS pH 6.1 (*p* = 1.1e−14) and proteolysis (*p* = 2.9e−23). TRS pH 6.1 showed significant difference compared with proteolysis (*p* = 2.9e−03) at an alfa level of 0.02 (Table 2, Fig. 5).

In addition, the three LNA microRNA probes provided high specificity; no cross-hybridization of any of the three LNA microRNA probes was observed, and as expected, no staining of the LNA miR-205-5p probe was observed in kidney and placenta tissue (Fig. 1).

No cross-hybridization with the scramble LNA probe or background staining was observed (Fig. 1). Prevalent expression was found for miR-205-5p in the basal compartment of epithelial cells in the hyperplastic prostate, tonsillar tissue, and cervix (both squamous epithelium); for miR-126-3p in endothelial cells of all tissue specimens; and for miR-145-5p in smooth muscle cells in arterial walls (all specimens) and in stromal smooth muscle cells of a hyperplastic prostate (Fig. 1).

### Consensus

Intraclass correlation coefficients (ICC) tests were performed for all probe staining intensities. The evaluations were performed independently by three raters resulting in an overall ICC of 0.75 indicating good interrater agreement. Hence, the closer the ICC is to one the better (Supplementary Table S5). By stratifying agreement into three categories based on periods of fixation, 5–24 h, 30–76 h, and 100–144 h of fixation led to a ICC of 0.58, 0.64, and 0.78, respectively. Focusing on rating microRNA retrieval, proteolysis, HmiRR using TRS pH 6.1 with or without pepsin and HmiRR using TRS pH 9 with or without pepsin had an ICC of 0.71, 0.77 and 0.75, respectively. All individual ICC for the ten treatments (ICC 1) and twelfth tissue-fixation categories (ICC 2) are displayed in Table 1.

### Additional results and remarks

**Figure 6:**
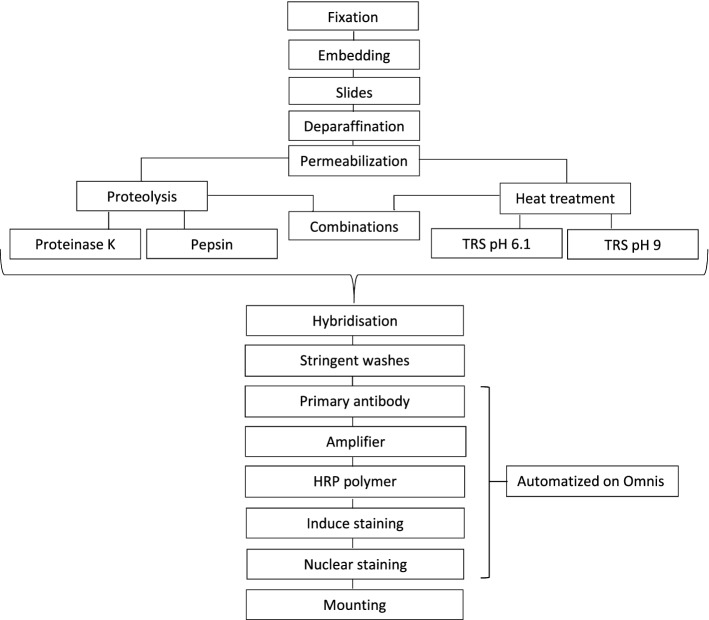
Flowchart of microRNA in situ hybridization. The first four boxes at the top indicate the standard procedure, including tissue fixation, tissue embedding (paraffin embedding), cutting of slides and permeabilization. Permeabilization is achieved with proteolysis, Heat-mediated microRNA Retrieval (HmiRR) or a combination of HmiRR followed by enzymatic treatment with pepsin. After permeabilization, all slides were subjected to the same procedure: hybridization and stringent washes. Application of primary antibody, amplifier, and horseradish peroxidase (HRP) polymer and nuclear staining could be automatized on a Dako Omnis platform. To preserve the tests, all slides were mounted at the end.

This protocol is a one-day protocol. If one prefers, after hybridization and stringent washes (Fig. 6), tissue sections can be stored overnight in Phosphate-buffered saline, PBS. Hence, continuing the protocol the following day (data not shown). Furthermore, all steps after stringent washes can be automatized using e.g. Dako Omnis IHC or other automated staining platform (Fig. 6). Additionally, according to preanalytic conditions, without postfixation with fixative 1-ethyl-3-[3-dimethylaminopropyl] carbodiimide (EDC), HmiRR with pH buffers (TRS pH 6.1/9) is compatible with other ISH methods such as fluorescence ISH or staining methods using NBT/BCIP (ready-to-use tablets, Roche, cat. no. 11 697 471 001 or equivalent), Levamisole (Fluka, cat. no. 31742 or equivalent) and Nuclear Fast Red nuclear counterstain (Vector Laboratories, cat. no. H-3403 or equivalent) (Supplementary Figure S2). Images of miR-205-5p probe staining in mammary gland is also displayed in Supplementary Figure S2. Subsequently, ISH using HmiRR with an e.g. high pH buffer can be performed, if one wish to combine detection of a specific target microRNA with antigenic epitopes (immunohistochemistry), because permeabilization using HmiRR does not alter antigen epitopes as protease treatment does. A long fixation time provides better results for long-term storage of FFPE tissues. Permeabilization and antigen retrieval with a pH buffer provide better detection conditions if one wishes to examine FFPE tissue samples after long-term storage.

## Discussion

Target microRNA preservation and accessibility are prerequisites for having a robust standardized microRNA-ISH assay of formalin-fixed and paraffin-embedded tissue. Previous attempts to improve microRNA hybridization efficiency or to increase the sensitivity of the microRNA-ISH assay, especially for low abundance microRNA types, have emphasized the difficulties and the need for optimization when working with microRNA and ISH, regardless of whether commercial kits are used^8,22–24^. Furthermore, not all commercial kits are compatible if one wants to demonstrate co-located proteins and microRNAs via direct or indirect detection^19,22^.

There are different difficulties when working with microRNA and ISH. Formalin fixation conditions, such as concentration, time or temperature, influence successful detection of microRNA. Additionally, conditions for permeabilization differ between tissues; hence, some tissues undergo strong permeabilization due to lower binding accessibility of the probe to the microRNA. E.g., target microRNA in lymphocytes require a more gentle pretreatment approach compared to detection of target microRNA in squamous epithelium. Moreover, the thickness of FFPE sections is an important parameter in choosing a sufficient microRNA retrieval method, and typically, more gentle pretreatment regimes are required when applied on thin sections^21^.

Overdigestion is one of the main issues when working with protease treatment (Figs. 2, 4). Therefore, we used a “test battery approach” involving HmiRR to search for a pretreatment method that would generate robust assays results (Tables 1, 2). Our approach provides beneficial knowledge regarding preanalytical parameters. For reproducibility purposes, Supplementary Figure S3 demonstrates results for HmiRR with TRS pH 9 without enzymatic treatment from the optimization phase and from the final study setup.

Our study is based on LNA probe detection of microRNA, since this type of probe is the most frequently used probe for small RNA ISH^24^, and was inspired by a study by Jørgensen et al.^25^. We showed that the ISH protocol could be conducted manually and could, if preferred, be semiautomated with the use of a Dako Omnis IHC and ISH platform and completed within 24 h (Fig. 6). It is also possible to break the one-day workflow into a two half-day setup in which only 4 h are required per day.

By comparing different control LNA microRNA probes, duration of fixation, and different permeabilization strategies across tissues, the overall best performing method was HmiRR in TRS pH 9 regardless of additional pepsin treatment (Table 1). In addition, a long fixation time was found to better preserve the microRNAs within tissues (Table 2).

We used 5 µm thick tissue sections, which is twice as thick as the size (2.5 µM) normally used for immunohistochemistry in the Department of Pathology, Zealand University Hospital, Roskilde, Denmark. A wide range of section thicknesses (4–40 µM) have been used in previous studies. None of the studies argues whether the chosen thickness of the paraffin sections matter, and none have tested the validity of different FFPE section thicknesses when working with microRNA^7,8,15,24–27^.

Previous studies have used proteinase K for permeabilization, applying various concentrations and incubation times (temperature with enzyme)^7,16,23,25,28^. Masuda et al. demonstrated that proteinase K was effective for retrieval of RNA even in tissue exposed to a longer fixation time in formalin and in archival samples. Masuda et al. stated that both proteinase K and heat treatment (Tris-EDTA buffer, pH 7.0) were able to reverse chemical modifications induced by formalin. However, suggestions for heating modifications have been made with regard to pH dependence, higher temperatures and longer incubation times^28^.

Nuovo et al. included an overnight in situ hybridization with labeled LNA probes and applied pepsin for pretreatment. Pepsin was preferred over proteinase K because the final outcome was less sensitive to the digestion parameters (e.g., concentration of the enzyme, time, and temperature) and rarely caused overdigestion of tissue^8^. Thus, low signal intensity after proteolysis treatment does not necessarily reflect low expression of the given microRNA but could also be a bias caused by the proteolysis treatment and lack of appropriate microRNA retrieval. Hence, examination of morphology is equally important. If proteolysis is insufficiently applied for too short a time, low signal intensity can be observed. However, the general recommendation by Nuovo et al. was to use proteinase K for permeabilization of tissue that has been fixed for days or weeks in formalin. Due to the overdigestion tendency of proteinase K, Nuovo et al. recommended that the digestion time be reduced to 5–10 min^8^. Similarly, a study by Jørgensen et al. recommended that proteinase K digestion be limited to 8 min^25^. However, we found proteinase K to be inferior to HmiRR across tissue types, especially with shorter fixation times.

In general, there is not a “standardized” procedure for applying enzymatic digestion for retrieval of microRNA, and the proteolytic step needs to be adjusted accurately according to both the length of fixation in formalin and the type of tissue. In this regard, HmiRR seems more consistent but still requires that a pretreatment “test battery approach” be performed for each individual LNA microRNA probe to be optimized.

Other studies have employed HmiRR: Chaudhuri et al. (buffer pH = 6.4, for 40 min at 90 °C)^15^ and de Planell-Saguer et al. (buffer pH = 6.0, for 10 min)^16^. The study by de Planell-Saguer et al. did not report whether exposure of tissue sections to longer pretreatment with citrate buffer at pH 6 provided better permeabilization than a 10 min exposure^16^. In our study, pretreament procedures based on TRS pH 6.1 gave inferior results compared to TRS pH 9-based procedures under the same boiling conditions: 20 min at 100 °C (Table 1).

In general, HmiRR, especially in TRS pH 9, achieved stable retrieval of microRNA across tissue types and fixation durations. Addition of pepsin for 3 min seemed equally effective. Increasing the pepsin exposure time to 5 min did improve staining intensity slightly but not statistic significant (Table 1, Supplementary Table S4). Surprisingly, a longer fixation time in formalin increased the intensity of the microRNA signal (Fig. 3, Tables 1, 2). This observation conflicts with other studies in which ISH was applied for detection of RNA or DNA, showing that formalin fixation longer than 24–36 h can cause problems^21^. One can speculate whether tissue exposed to a long formalin fixation time has the ability to retain a larger amount of diffusible small RNA transcripts (e.g., microRNA), providing better detection with ISH using short LNA probes, whereas larger probes are obstructed in targeting RNA/DNA sequences due to poor penetration through tissue exposed to long term fixation that leads to heavy cross-linking. This would be in line with our findings.

Several studies have found it beneficial to include a postfixation step with 1-ethyl-3-[3-dimethylaminopropyl] carbodiimide (EDC), EDC-hydrochloride (EDC-HCl) and 5-ethylthio-1H-tetrazole (5-ETT) in 1-methylimidazole buffer as a fixative water-soluble condensation reagent for better microRNA crosslinking^7,16,27^. In contrast, a study by Jørgensen et al. was unable to improve the effect when adding the EDC step^25^. Nevertheless, microRNA fluorescence in situ hybridization (FISH) and conventional immunohistochemical (IHC) methods are incompatible with ISH EDC postfixation^27^. Therefore, a postfixation step was excluded in this approach.

This microRNA detection approach is applicable to fluorescence ISH staining or staining methods using NBT/BCIP (Roche), Levamisole (Fluka) and Nuclear Fast Red nuclear counterstain (Vector Laboratories) (Supplementary Figure S2). Our approach, along with the finding that HmiRR in TRS pH 9 with or without pepsin addition retrieved the selected microRNAs in this study, is compatible with the vast majority of immunohistochemical markers typically requiring Heat-Induced Epitope Retrieval (HIER) in the same buffer (TRS pH 9). In fact, in immunohistochemical external quality assurance programs, such as UK NEQAS and NordiQC, virtually all markers assessed require HIER for optimal performance^29,30^. This questions the use of proteases for retrieval of antigenic epitopes. Hence, setting up a combined method should be considered when working with microRNA detection.

## Conclusion

HmiRR using TRS pH 9 with and without pepsin digestion provides robust results, with the best microRNA hybridization efficiency after long tissue fixation in formalin (range 100–144 h).

The method described herein provides a quick and easy protocol for detection of microRNA using ISH, even for an unexperienced user. This protocol can be run within one day or be divided into two half-work days. Additionally, it is possible to convert the workflow into a semi-automated protocol (Fig. 6). Although, HmiRR is stable and effective in our setting, different tissues and other microRNAs might require different approaches. This is why the battery test approach for different pretreatment strategies should be conducted every time a new test is optimized.

In the context of multiplexing involving detection of microRNAs, HmiRR using an appropriate buffer (e.g., TRS pH 9) might be more beneficial than proteolysis because only few antigenic epitopes (IHC) require enzymatic treatment for optimal performance.

### Perspectives

The development of a robust protocol for microRNA ISH is likely to find wide application in both clinical- and research-related contexts. The use of a pretreatment test battery on a multiblock is only a part of the first validation process, identifying an optimal retrieval protocol for a given microRNA of interest and providing consistent signal intensity and reproducible results. Our results indicate that a longer fixation time in formalin provides better and more intense signals, which contradicts the findings of other studies and therefore warrants further investigation. However, once the optimal microRNA retrieval protocol has been identified, further validation is needed, particularly for clinical purposes. Thus, if microRNA ISH can be applied for detection of disease-related microRNAs in both patient and normal tissues, that would be helpful to evaluate the method for future clinic application. Validation should be conducted using relevant clinical material displaying a wide range of microRNA densities (both negative and positive cases), and thereby, the sensitivity and specificity of the microRNA ISH protocol(s) can be accurately determined. Ideally, these results should be compared with those produced using other methods, such as cell lines known to express the target microRNAs, to confirm the robustness and specificity of the optimized microRNA ISH assay.

## Material and reagents

Equipment:

1. Microtome, Microm HM 355S, Thermo Fisher
2. Cold light source, SCHOTT KL200, SCHOTT AG
3. Staining dish according to Hellendahl, DURAN, code no. SCOT233150002, DWK Life Sciences, VWR, VWR International, LLC.
4. Staining jars, DURAN, Staining dishes acc. to Schiefferdecker, for up to 10 slides (with slide rack), code no. SCOT233160003, DWK Life Sciences, VWR, VWR International, LLC.
5. Cover slips, thickness 1, D 263 M colorless borosilicate glass, 20 mm × 20 mm, code no. BB02000-200A153MNT0, Thermo Scientific, Thermo Fisher Scientific
6. FLEX IHC Microscope Slides, code no. K8020, Dako, Agilent Technologies, Inc.
7. Metal tweezers, USBECK Laborgeräte, code no. 232-0088, VWR International, LLC.
8. Dako Hybridizer, Dako, Agilent Technologies, Inc.
9. Pasteur pipettes, disposable, LDPE, SEMADENI, code no. SEMA4271, VWR International, LLC.
10. Fisherbrand Free-Standing Microcentrifuge Tubes with Screw Caps, code no. 12330433, Fisher Scientific, Thermo Fisher Scientific Inc.
11. AccuBlock Digital Dry Bath, code no. D1302, Labnet International, Inc.
12. Light Microscope, Nikon Eclipse 80i, Nikon Instruments, Inc.
13. Microwave oven (MWO) with 6th Sense technology from Whirlpool
14. DAKO Omnis ICH platform, Agilent Technologies, Inc.

Reagents for dewaxing and retrieval of microRNA:

1. Xylenes, histological grade, code no. 534056, Sigma-Aldrich, Inc.
2. Ethanol absolute ≥ 99.8%, AnalaR NORMAPUR ACS, Reag. Ph. Eur. analytiskt reagens, VWR Chemicals, code no. 20821.296, VWR International, LLC.
3. Ethanol 96%, code no. 1680643, Kemetyl A/S
4. EnVision FLEX Wash Buffer (20x) (pH 7.6) code no. K8007, DM831, Dako, Agilent Technologies, Inc. Tris-buffered saline solution containing Tween 20, pH 7.6 (± 1)
5. EnVision FLEX Target Retrieval Solution (TRS), pH 9, (10x) code no. S2367, Dako, Agilent Technologies, Inc.
6. EnVision FLEX Target Retrieval Solution (TRS), low pH (pH 6.1), (50x), code no. K8005, DM829, Dako, Agilent Technologies, Inc.
7. MicroRNA ISH Optimization Kit 8 (FFPE), Exiqon Inc. code no. 339457, Qiagen: Proteinase K solution, Lyophilized, 12 mg, 1.25 mL, Exiqon Inc., Denmark
8. Pepsin solution, Ready-to-use, code no. ES-0001, ZytoVision GmbH, Germany

Hazard notes: perform steps with Xylene and Ethanol in a fume hood with appropriate protective gear. Wear protective gloves and eye/face protection, when working with proteinase K, pepsin solution, EnVision FLEX Wash Buffer (20×) and Target Retrieval solutions.

Reagents for hybridization:

1. Nuclease-free water (not DEPC-Treated), Invitrogen, code no. AM9932, Thermo Fisher Scientific Inc.
2. 2× miRNA ISH buffer 25 mL, code no. 90-012, Exiqon Inc., Denmark
3. Fixogum, code no. 11FIXO0050, MP Biomedicals
4. Phosphate-buffered saline (PBS) tablet, code no. 79382-50TAB, Sigma-Aldrich, Inc.
5. 20× Sodium-Saline Citrate (SSC) buffer stock solution, Ultrapure, code no. 15557044, Invitrogen, Thermo Fisher Scientific Inc.
6. MicroRNA ISH Optimization Kit 8 (FFPE), Exiqon Inc., code no. 339457, Qiagen: labeled Digoxigenin (DIG) Probe (miR-205-5p) conc. 25 µM. Scramble LNA Negative Control Probe (double DIG) 25 µM, 40 µL
7. Double labeled DIG Probe miR-126-3p, 10 nmol, Exiqon Inc., code no. 339112, Qiagen
8. Double labeled DIG Probe miR-145-5p, 10 nmol, Exiqon Inc., code no. 339112, Qiagen

Hazard note: wear protective gloves, when working with DIG probes.

Reagents for probe detection:

1. Dako Pen, code no. S2002, Dako, Agilent Technologies, Inc.
2. EnVision FLEX Wash Buffer (20×) (pH 7.6), Tris-buffered saline solution containing Tween 20, pH 7.6 (± 1) code no. K8007, DM831, Dako, Agilent Technologies, Inc.
3. Mouse-anti-DIG, Ready-to-use, code no. AB-0001-30, ZytoVision GmbH, Germany
4. EnVision FLEX+ Peroxidase-Blocking Reagent, ready-to-use (15 mmol/L), code no. SM801, Dako, Agilent Technologies, Inc.
5. EnVision FLEX+ Mouse High pH, (Link), code no. K8002, DM804, Dako, Agilent Technologies, Inc.
6. EnVision FLEX Substrate Working Solution: 1 drop EnVision Flex DAB+ Chromogen (DM827) per 1 mL EnVision FLEX Substrate Buffer (SM803), Dako, Agilent Technologies, Inc.
7. Nuclease-free water (not DEPC-Treated), Invitrogen, code no. AM9932, Thermo Fisher Scientific Inc.
8. EnVision FLEX+ Haematoxylin, (Link), code no. K8008, SM806, Dako, Agilent Technologies, Inc.
9. PERTEX, mounting medium, Leica Microsystems, code no. LEIC811, VWR International, LLC.

Hazard note: if probe detection steps are not automatized, perform these steps in a fume hood with appropriate protective gear.

## Methods

### Study design

This protocol is for a one-day experiment. The first part of the protocol concerns pretreatment, denaturation and hybridization. The second part of the protocol involves detection and visualization using an anti-DIG reagent (AB-0001-30, ZytoVision GmbH, Germany), a mouse linker (DM804, EnVision FLEX+, Dako, Agilent Technologies, Inc.), a detection system (K8002, EnVision FLEX+ Mouse High pH, Dako, Agilent Technologies, Inc.) and the horseradish peroxidase (HRP) substrate 3,3-diaminobenzidine (DAB) (DM827, EnVision, Dako, Agilent Technologies, Inc.). Three probes with known hybridization patterns were chosen from optimization kits (339457, 339112, Exiqon Inc. Qiagen): miR-126-3p, miR-145-5p and miR-205-5p. These three microRNAs were used as positive controls. A scramble probe was used as a negative control (339457, Exiqon Inc. Qiagen). Kidney tissue was included in the multiblock as another negative control, because miR-205-5p is not expressed in the kidney, hence no expression of miR-205-5p was expected. The following expression patterns were expected; miR-126-3p is normally expressed in endothelial cells (all specimens), miR-145-5p in smooth muscle cells (all specimens) and miR-205-5p in squamous epithelium (predominantly the basal compartment) of the tonsil and cervix and in basal epithelial cells of the hyperplastic glands of the prostate^20,25,29^. Because the aim was to design a protocol suitable for a routine laboratory workflow, RNAse Zap treatment of the equipment was not conducted. All experiments were performed in a normal standardized clean laboratory environment. The workflow is presented in Fig. 6. Images using this approach can be found in Figs. 1 and 4. Additionally, we verified that pH buffer treatment is compatible with other in situ hybridization methods: ISH and FISH (Supplementary Figure S2). These applicable methods were beyond the aim of this study but have been emphasized as an issue elsewhere^27^. Notable, the series of preanalytic treatment have been tested together once. For demonstrating reproducibility, Supplementary Figure S3 displays images of HmiRR with TRS pH 9 without enzymatic treatment from the optimization phase of the study.

### Preparation and pretreatment

All tissue specimens were fully anonymized, and consequently the study did not require scientific ethics approval. Fresh tissue samples from the cervix, kidney, tonsil, placenta and hyperplastic prostate were sliced into 10 × 10 × 3 mm pieces, fixed for different time periods in 10% buffered formaldehyde (formalin) solution (range 5–144 h) and processed into paraffin using standard procedures. A multiblock was constructed by punching out relevant tissue cores (4 mm) from the paraffin-embedded tissue, and detailed information regarding fixation time can be seen in Supplementary Figure S1. The multiblocks were cut (5 µm), and slides were air-dried overnight, heated for 1 h at 60 °C, dewaxed in xylene (534056, Sigma-Aldrich, Inc.) (3 × 5 min), rehydrated through a graded alcohol (20821.296, VWR International, LLC., 1680643, Kemetyl A/S) series (99.9%, 96%, and 70% ethanol; each 3 × 2 min), and finally placed in nuclease-free water for 5 min. All glassware (staining jars and dishes (SCOT233150002, SCOT233160003, VWR International, LLC.), cover glasses (BB02000200A153MNT0, Thermo Fisher Scientific), and object glasses (K8020, Dako, Agilent Technologies, Inc.) and metal tweezers (232-0088, VWR International, LLC.) were used under the same hygiene standard applied in a hospital. Object glasses and cover glasses were taken from unopened packages.

The study included ten different combinations of microRNA retrieval methods to compare efficiency, robustness and the quality of signal intensity.

### Pretreatment with proteolysis

Proteinase K buffer (339457, Exiqon Inc. Qiagen) was diluted according to the manufacturer’s instructions and bought to 37 °C. Slides receiving proteolysis were treated for different intervals (range 2.5–20 min).

Pepsin was only used for slides receiving HmiRR. The pepsin solution was in a ready-to-use format (concentration not stated by the manufacturer). Slides were first subjected to heat treatment in an MWO for 20 min at 100 °C using an appropriate buffer, cooled for 10 min at room temperature (RT), and washed with DI water before application of the pepsin solution (ES-0001, ZytoVision GmbH, Germany) for 3 or 5 min. After incubation with pepsin, all slides were washed for 5 min in PBS (79382-50TAB, Sigma-Aldrich, Inc.) and RNase-free water (AM9932, Thermo Fisher Scientific Inc.).

### Pretreatment using Heat-induced microRNA Retrieval

Slides were subjected to heat treatment in an MWO for 20 min at 100 °C using either TRS pH 6.1 (DM829, Dako, Agilent Technologies, Inc.) or TRS pH 9 (S2367, Dako, Agilent Technologies, Inc.), cooled for 10 min at room temperature and washed for 5 min in PBS (79382-50TAB, Sigma-Aldrich, Inc.) and RNase-free water (AM9932, Thermo Fisher Scientific Inc.).

### In situ hybridization, microRNA

A MicroRNA ISH Optimization Kit 8 (FFPE) (339457, Exiqon Inc. Qiagen) was used for in situ hybridization targeting the microRNA miR-205-5p. Probes for miR-126-3p and miR-145-5p were also included (339112, Exiqon Inc. Qiagen). Before use, all probes were denatured at 90 °C for 4 min. For the hybridization mix, the probe concentration was 120 nM in 1:1 nuclease-free water mixed with 2× microRNA ISH buffer. Hybridization mix (25 µL) was added onto each of the slides, which were then coverslipped (BB02000200A153MNT0, Thermo Fisher Scientific) and sealed with fixogum (11FIXO0050, MP Biomedicals). Slides were hybridized for 1.5 h at 55 °C using a hybridizer (Dako Hybridizer, Dako Agilent). The fixogum was removed and the slides were placed in 2× SSC (15557044, Thermo Fisher Scientific Inc.) at room temperature. All slides received stringent washes and were subjected to 2× SSC for 1 min at 57 °C, 0.2× SSC buffer for 20 min at 57 °C and 0.2× SSC for 5 min at room temperature. All SSC solutions were made fresh from a 20× SSC stock solution (15557044, Thermo Fisher Scientific Inc.). Finally, slides were placed twice in PBS (79382-50TAB, Sigma-Aldrich, Inc.) for 3 min.

### Automated microRNA probe detection on a Dako Omnis IHC platform

After hybridization, the slides were loaded on a Dako Omnis IHC platform (wet program) (Dako Omnis, Agilent Technologies, Inc.), and probe reactions were detected using an anti-DIG antibody (AB-0001-30, ZytoVision GmbH, Germany) and visualized using a standard EnVision FLEX+/DAB system (DM827, SM803, Dako, Agilent Technologies, Inc.). Briefly, the slides were incubated with a mouse anti-DIG primary antibody (AB-0001-30, ZytoVision GmbH, Germany) for 30 min followed by blocking of endogenous peroxidase activity using EnVision FLEX Peroxidase-Blocking Reagent (SM801, Dako, Agilent Technologies, Inc.) for 3 min. Reactions were detected using EnVision FLEX+ (DM804, K8002, Dako, Agilent Technologies, Inc.) and visualized using EnVision FLEX Substrate Working Solution (DM827, SM803, Dako, Agilent Technologies, Inc.) according to the recommendations provided by the manufacturer. The incubation temperature was 32 °C, and between all incubation steps, the slides were rinsed with wash buffer (DM831, Dako, Agilent Technologies, Inc.). Finally, the slides were incubated and counterstained with hematoxylin solution (SM806, Dako, Agilent Technologies, Inc.) for 3 min, rinsed in tap water, dehydrated using 3× 99% ethanol (20821.296, VWR International, LLC.) and permanently mounted using pertex (LEIC811, VWR International, LLC.).

## Evaluation

The results were evaluated blinded for probe staining intensity and preservation of morphology and scored independently (Supplementary Figure S1 and Supplementary Table S1–S3). One person blinded all slides for three other raters to assess the results. For each probe, the grading scale was set by assigning the most successful test as the upper limit (numeric 3) and clear negative staining as the lowest value (0). Thus, tests equally successful as the best rated can be assigned 3. Evaluation scale: 0 = no signal intensity; 1 = weak signal intensity; 2 = moderate signal intensity; 3 = strong signal intensity. An asterisk * was given if the morphology of the tissue structures was reported impaired by a rater. Slides were examined with a light microscope (Nikon Eclipse 80i) with optical zoom at 100×, 200× and 400× magnification. Both the overall impression and the local microRNA expression site were taken into account.

### Statistics

All statistical analyses and corresponding graphic outputs were conducted with the statistical software R version 4.0.2. The following packages were used: readxl version 1.3.1, dplyr 1.0.2, ggplot2 version 3.3.2, irr version 0.84.1, ggthemes version 4.2.0, ggpubr version 0.4.0. Inspection of skewness was done by generating qq-plots for the quantiles using qqnorm() function (data not shown). A Shapiro–Wilk’s test was used for statistical testing for normality. To reduce skewness of data, data was log-transformed. For pairwise comparison of data, a non-parametric paired Wilcoxon rank sum test was used, with *p* value adjustment using the Benjamini–Hochberg procedure using False-discovery rate. The statistical alpha level was set according to Bonferroni correction depending on the amount of tests performed. Intraclass correlation coefficients (ICC) test was performed using the icc() R function. For the icc() function: model was set to “twoway”, type were set to “consistency” and unit were set to either “single” or “average”, dependent on the type of observation in the given test.

### Consensus

The intensity for each probe (miR-126-3p, -145-5p and -205-5p) was assessed on the basis of seven gradings (0, 0.5, 1, 1.5, 2, 2.5, 3). ICC test was used to compare statistical agreement among raters. ICC test was performed for the intensity rating for fixation and for permeabilization. For all evaluations, an overall ICC test was calculated. Individually agreement on the ten pretreatments and the twelfth tissue-fixation categories was assessed.

### Ethics

None of the authors has any conflicts of interest to declare. This project was conducted on anonymized patient material. The patient material consist of tissue biopsies taken from surgical specimens (cervix, kidney, placenta, prostate, and tonsil tissues). All specimens were surgically removed for treatment or diagnostic purposes. According to Danish law, completely anonymized left over material from diagnostic procedures can be used for research without patient consent or specific approval^31^. This study received funding from The Danish Rheumatism Association (Project No.: R142-A4194 and R144-A4195).

## Supplementary Information

Supplementary Informations.
