# Supplementary material for: A novel approach for microRNA in situ hybridization using locked nucleic acid probes
Source: Sci Rep. 2021 Feb 24;11:4504. doi: 10.1038/s41598-021-83888-5 (PMC7904755; doi:10.1038/s41598-021-83888-5)
Supplement: Supplementary file 1 — Supplementary Informations. [file 41598_2021_83888_MOESM1_ESM.pdf]

A novel approach for microRNA in situ hybridization using locked nucleic acid probes

Authors: Isabella W. Paulsen[1.2.¶]. Michael Bzorek[3]. Jesper Olsen[2.4]. Birgitte Grum-Schwensen[1]. Jesper T. Troelsen[2]. Ole B. Pedersen[1. ¶].

1 Department of Clinical Immunology. Zealand University Hospital. Koege. Denmark.

2 Department of Science and Environment. Roskilde University. Roskilde. Denmark.

3 Department of Pathology. Zealand University Hospital. Roskilde. Denmark.

4 Department of Surgery. Zealand University Hospital. Koege. Denmark.

¶ Corresponding authors: iwp@regionsjaelland.dk/ olbp@regionsjaelland.dk

Isabella W. Paulsen. MSc. / Ole Birger Pedersen. associate professor. chief physician

Department of Clinical Immunology. Zealand University Hospital. Koege.

Ringstedgade 77B. DK-4700 Naestved. Denmark.

Supplementary content

Supplementery Figure S1
Supplementery Figure S2
Supplementery Figure S3
Supplementery Table S1
Supplementery Table S2
Supplementery Table S3
Supplementery Table S4
Supplementery Table S5

Supplementary Figure S1

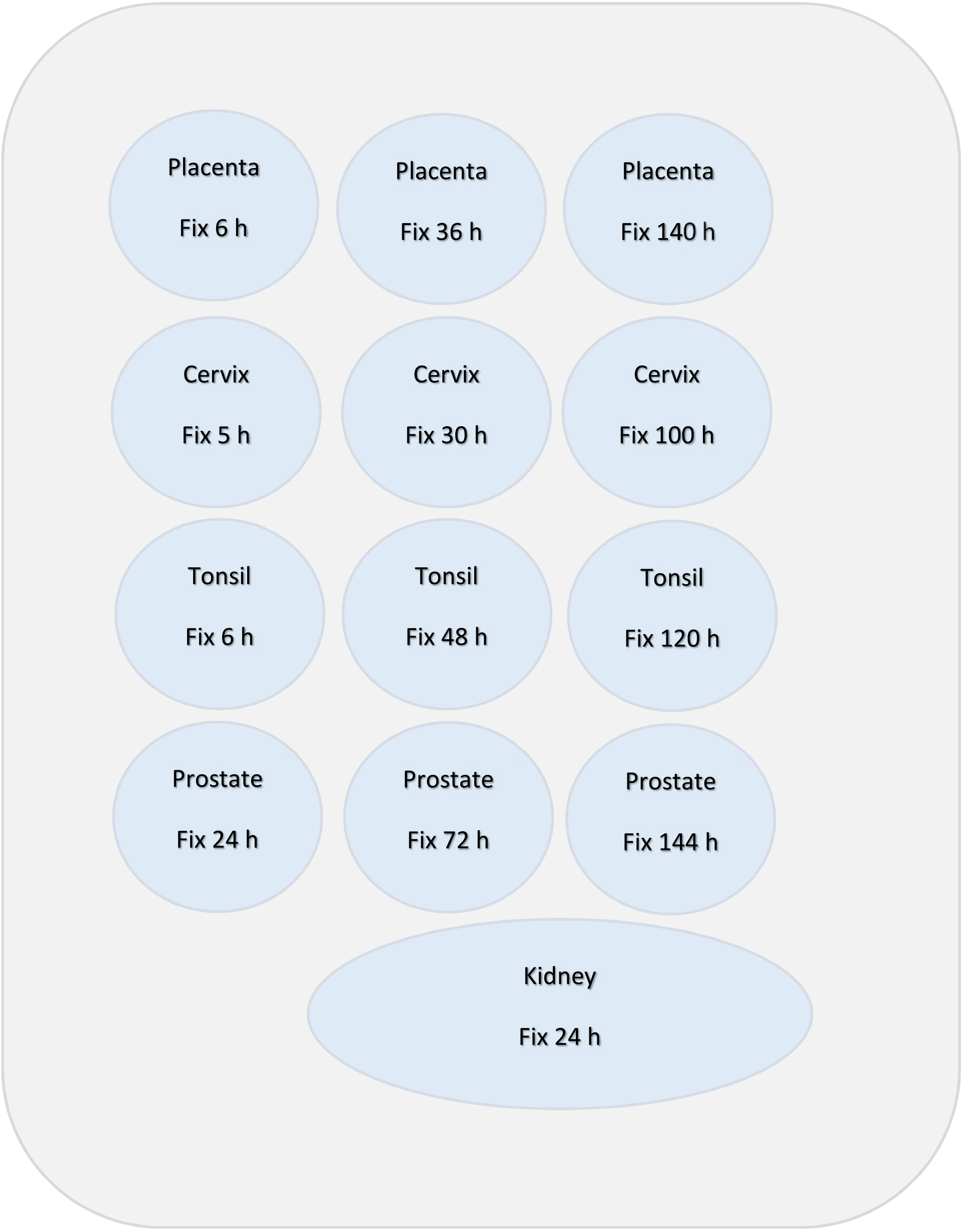

Supplementary Figure S1 – Multiblock of formalin-fixed paraffin-embedded tissue samples. A multiblock was constructed as a test battery by punching out relevant tissue cores (4 mm) from the paraffin-embedded tissue, which had been formalin fixed. Detailed information regarding tissue type and fixation time (hour, h) is found in each circle. First row: placenta tissues. Second row: cervical tissues. Third row: tonsil tissues. Fourth row: prostate tissues. First column: tissue fixed for a short time, 5-24 hours. Second column: tissue with moderate fixation, 30-72 hours. Third column: tissue with long fixation, 100-144 hours. Bottom: negative control, kidney tissue fixed for 24 hours.

Supplementary Figure S2

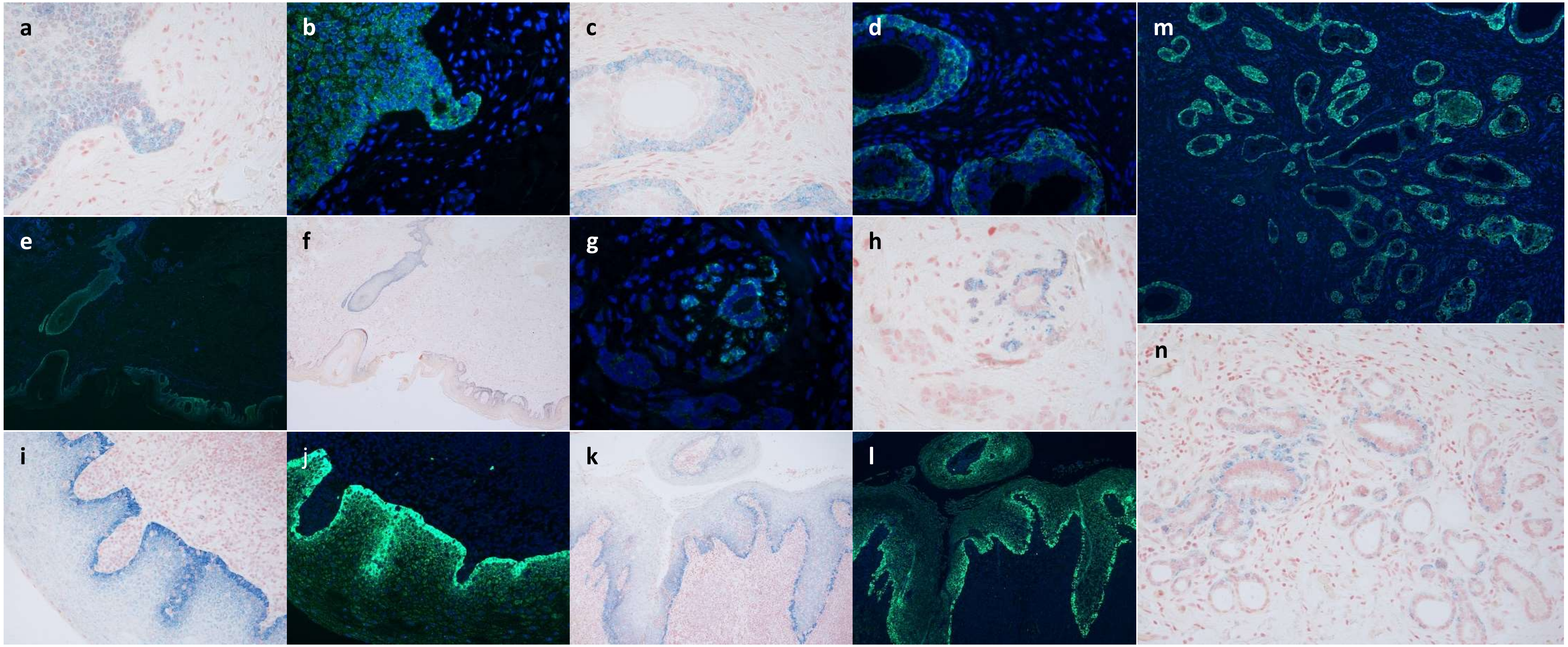

Supplementary Figure S2 – Applicable in situ hybridization methods. The images demonstrates Fluorescence in situ hybridization, FISH (Black background) and in situ hybridization, ISH using NBT/BCIP (Roche), Levamisole (Fluka) and Nuclear Fast Red nuclear counterstain (Vector Laboratories) (Light background). All images illustrate miR-205-5p probe staining (Blue staining in ISH and fluorescence green in FISH). Nuclear counterstaining appears red in ISH and blue in FISH. Skin tissue: (**a-b**) and (**e-f**). Tonsil tissue: (**i-l**). Prostate tissue: (**c-d**) and (**m**). Mammary Gland: (**g-h**) and (**n**). Optic zoom at magnification: 40x (**e-f**), 100x (**k-m**), 200x (**j** and **n**) and 400x (**a-d**) and (**g-i**).

Suplementary Figure S3

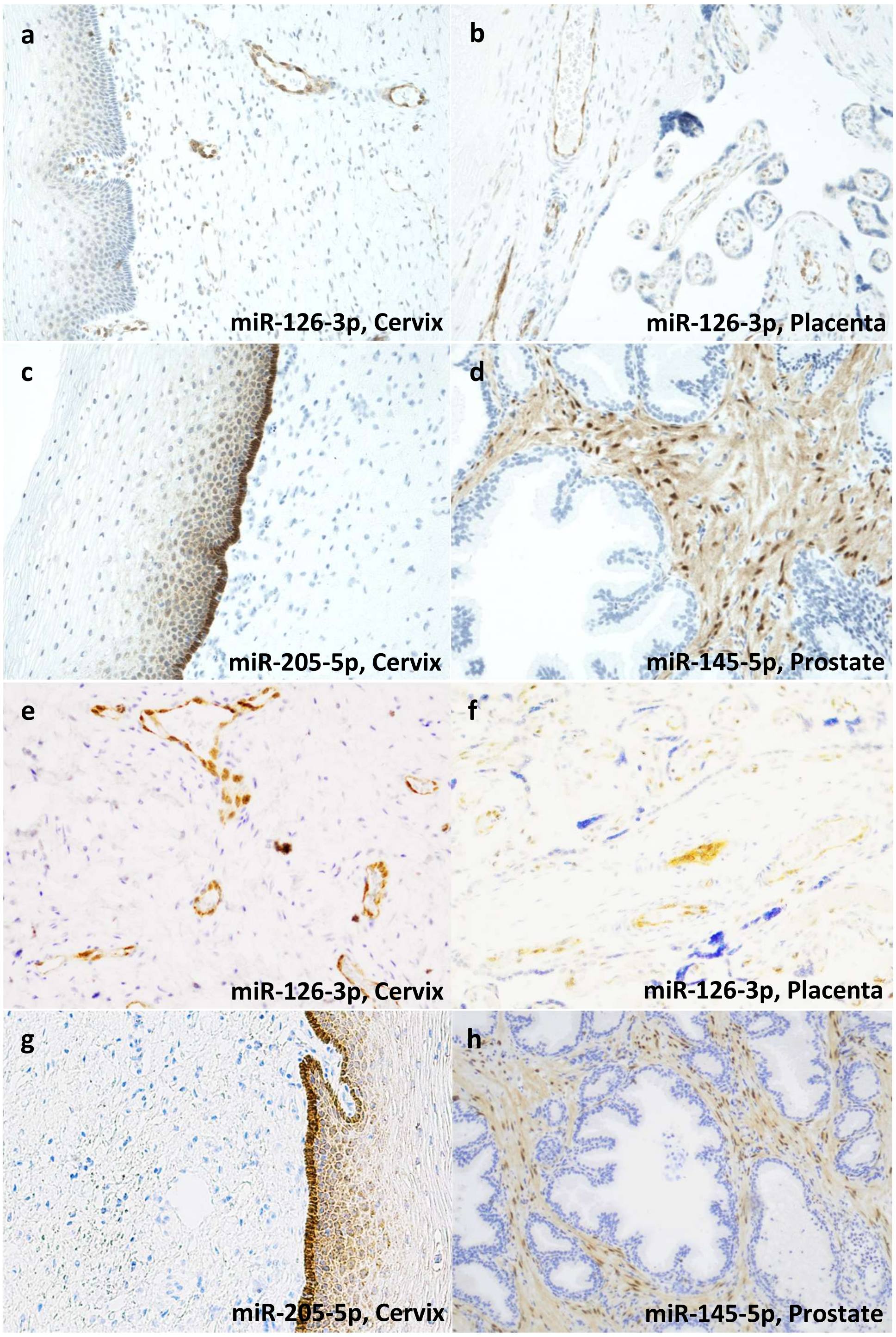

Supplementary Figure S3 – In situ hybridization (ISH) reproducibility images. All pictures were taken at magnification 200x optic zoom with light microscopy, except image (**h**) (100x). All pictures display heat-induced microRNA Retrieval using Target Retrieval Solution pH 9 (20 minutes at 100°C) on prostate, placenta and cervical (cervix) tissue fixed in formalin for variable lengths of time (100-144 hours). Brown staining indicates a positive target probe signal, blue indicates negative signal in the remaining cells, hence background staining. MicroRNA restriction: miR-126-3p is stably expressed in endothelial cells; miR-145-5p is stably expressed in smooth muscle cells; miR-205-5p is stably expressed in the basal compartment of epithelial cells. Probe staining: miR-126-3p (**a**-**b**) and (**e**-**f**), miR-205-5p (**c**) and (**g**) and miR-145-5p (**d**) and (**h**). Cervix: (**a**), (**c**), (**e**) and (**g**). Placenta: (**b**) and (**f**). Prostate: (**d**) and (**h**).

Supplementary Table S1

Treatment Hsa-miR-126-3p	Time (mins)	Cervix			Prostate			Tonsil			Placenta		
Fixation time (hours)		5	30	100	24	72	144	6	48	120	6	36	140
Proteinase K	2.5	0.0	0.3	0.8	0.0	0.3	1.0	0.0	0.5	1.7	0.0	0.0	1.2
Proteinase K	5	*0.0	*0.0	*0.0	*0.0	0.2	0.5	*0.0	0.0	0.0	*0.0	*0.0	0.0
Proteinase K	10	*0.5	*0.8	*1.3	*0.7	0.8	1.7	*0.5	*1.0	*1.8	*0.0	*0.0	*0.8
Proteinase K	20	*0.3	*0.2	*0.2	*0.0	*0.0	*0.2	*0.2	*0.0	*0.0	*0.0	*0.0	*0.0
TRS pH 6.1	20	0.3	0.7	2.2	0.2	0.2	1.2	0.2	1.0	2.0	0.2	0.3	1.2
TRS pH 6.1 + pepsin	20 + 3	0.3	0.7	1.8	0.2	0.5	1.2	0.5	0.8	2.0	0.0	0.2	1.3
TRS pH 6.1 + pepsin	20 + 5	0.7	1.0	2.7	0.3	0.7	2.2	0.7	1.2	2.5	0.3	0.3	1.8
TRS pH 9	20	1.2	1.3	3.0	0.8	1.5	2.7	1.5	1.7	2.7	0.3	0.8	2.0
TRS pH 9 + pepsin	20 + 3	1.0	1.5	3.0	0.7	1.3	3.0	1.7	2.3	3.0	0.5	1.3	2.3
TRS pH 9 + pepsin	20 + 5	1.3	1.8	3.0	0.5	1.3	2.0	1.0	2.3	3.0	0.3	1.0	2.7

Supplementary Table S1 – Systematic overview of in situ hybridization experiments with a locked nucleic acid probe for miR-126-3p. The upper horizontal bar shows included tissues, and the second upper horizontal column indicates the time of fixation in formalin. The vertical bar to the left indicates the pretreatment of choice: proteinase K, heat-induced microRNA retrieval with or without pepsin addition, Target Retrieval Solution (TRS) pH 6.1 or TRS pH 9. The second vertical column shows the pretreatment duration time. + = weak signal intensity; ++ = moderate signal intensity; +++ = strong signal intensity; ( ) = no signal intensity; (+) = a half rated +; * = poor morphology has been reported by a rater.

Supplementary Table S2

Treatment Hsa-miR-205-5p	Time (mins)	Cervix			Prostate			Tonsil			Placenta		
Fixation time (hours)		5	30	100	24	72	144	6	48	120	6	36	140
Proteinase K	2.5	0.2	1.5	1.8	1.0	1.7	2.5	0.5	1.7	2.5	-	-	-
Proteinase K	5	*0.0	*0.0	*0.7	*0.2	*0.0	*0.8	*0.0	*0.2	*0.7	-	-	-
Proteinase K	10	*0.5	1.8	1.5	1.2	1.7	2.8	*0.8	2.2	1.8	-	-	-
Proteinase K	20	*0.2	*0.8	*1.5	*0.5	*1.0	*1.8	*0.2	*0.7	*1.8	-	-	-
TRS pH 6.1	20	0.5	0.8	1.7	1.2	2.2	3.0	0.8	1.7	2.3	-	-	-
TRS pH 6.1 + pepsin	20 + 3	0.3	1.0	1.5	1.3	2.2	3.0	1.2	2.2	2.7	-	-	-
TRS pH 6.1 + pepsin	20 + 5	0.7	1.3	2.0	1.3	2.2	3.0	0.8	1.5	2.5	-	-	-
TRS pH 9	20	2.2	2.3	3.0	1.8	2.2	3.0	1.7	2.3	3.0	-	-	-
TRS pH 9 + pepsin	20 + 3	1.3	1.7	2.5	2.0	2.2	3.0	1.3	1.7	2.5	-	-	-
TRS pH 9 + pepsin	20 + 5	1.7	2.3	3.0	2.2	2.3	3.0	1.7	2.7	3.0	-	-	-

Supplementary Table S2 – Systematic overview of in situ hybridization experiments with a locked nucleic acid probe for miR-205-5p. The upper horizontal bar shows included tissue, and the second upper horizontal column indicates the time of fixation in formalin. The vertical bar to the left indicates the pretreatment of choice: proteinase K, heat-induced microRNA retrieval with or without pepsin addition, Target Retrieval Solution (TRS) pH 6.1 or TRS pH 9. The second vertical column shows the pretreatment duration time. + = weak signal intensity; ++ = moderate signal intensity; +++ = strong signal intensity; ( ) = no signal intensity; (+) = a half rated +; * = poor morphology has been reported by a rater.

Supplementary Table S3

Treatment Hsa-miR-145-5p	Time (mins)	Cervix			Prostate			Tonsil			Placenta		
Fixation time (hours)		5	30	100	24	72	144	6	48	120	6	36	140
Proteinase K	2.5	0.7	0.8	2.3	2.3	2.5	3.0	*0.7	1.7	2.8	0.0	0.8	2.2
Proteinase K	5	*0.2	0.0	0.2	1.3	1.3	2.3	*0.0	0.0	0.3	0.0	0.0	0.0
Proteinase K	10	*0.8	1.7	3.0	2.3	2.5	3.0	*0.8	2.0	3.0	0.5	1.3	2.0
Proteinase K	20	*0.2	*0.3	*2.2	2.0	2.0	2.8	*0.3	*0.3	*2.2	*0.0	*0.8	*1.8
TRS pH 6.1	20	0.0	0.2	1.3	0.3	0.5	1.3	0.2	0.2	1.2	0.0	0.0	0.7
TRS pH 6.1 + pepsin	20 + 3	*0.7	*0.7	*1.8	0.7	1.2	1.7	*0.3	0.5	1.5	*0.0	0.2	1.5
TRS pH 6.1 + pepsin	20 + 5	*1.0	0.2	2.2	1.3	1.3	1.8	0.3	0.5	1.2	0.0	0.0	1.3
TRS pH 9	20	0.3	0.7	2.5	1.8	2.3	2.8	0.8	1.3	2.5	0.2	0.2	2.0
TRS pH 9 + pepsin	20 + 3	*1.8	0.7	3.0	1.7	1.8	1.8	*1.0	*1.3	2.2	0.2	0.0	2.3
TRS pH 9 + pepsin	20 + 5	1.3	0.7	2.8	1.5	2.0	2.8	*1.3	*1.5	*2.7	0.3	0.7	2.3

Supplementary Table S3 – Systematic overview of in situ hybridization experiments with a locked nucleic acid probe formiR-145-5p. The upper horizontal bar shows included tissue, and the second upper horizontal column indicates the time of fixation in formalin. The vertical bar to the left indicates the pretreatment of choice: proteinase K, heat-induced microRNA retrieval with or without pepsin addition, Target Retrieval Solution (TRS) pH 6.1 or TRS pH 9. The second vertical column shows the pretreatment duration time. + = weak signal intensity; ++ = moderate signal intensity; +++ = strong signal intensity; ( ) = no signal intensity; (+) = a half rated +; * = poor morphology has been reported by a rater.

Supplementary Table S4

	Proteinase K 2.5 min	Proteinase K 5 min	Proteinase K 10 min	Proteinase K 20 min	TRS pH 6.1	TRS pH 6.1 + pepsin 3 min	TRS pH 6.1 + pepsin 5 min	TRS pH 9	TRS pH 9 + pepsin 3 min
Proteinase K 5 min	1.1e-01								
Proteinase K 10 min	4.7e-06	5.9e-03							
Proteinase K 20 min	3.7e-16	1.7e-10	1.2e-04						
TRS pH 6.1	7.0e-04	1.3e-01	1.4e-01	4.7e-08					
TRS pH 6.1 + pepsin 3 min	3.3e-02	7.6e-01	3.8e-03	5.9e-12	1.4e-01				
TRS pH 6.1 + pepsin 5 min	6.4e-02	2.9e-01	2.1e-04	1.1e-12	8.6e-03	1.6e-01			
TRS pH 9	4.8e-02	7.5e-04	5.2e-09	6.8e-19	5.1e-07	5.9e-05	2.9e-03		
TRS pH 9 + pepsin 3 min	6.4e-02	1.0e-03	3.1e-09	1.6e-19	4.6e-07	5.9e-05	2.3e-03	8.1e-01	
TRS pH 9 + pepsin 5 min	8.0e-03	6.0e-05	1.0e-10	1.1e-20	1.3e-08	2.1e-06	5.0e-04	5.6e-01	4.2e-01

Supplementary Table S4 – Result table of pairwise comparison. Table displays Wilcoxon rank sum test for pairwise comparison of data. The upper horizontal bar and the first vertical bar to the left presents the pretreatments: proteinase K (2.5 – 20 minutes, min), Heat-induced microRNA Retrieval using Target Retrieval Solution (TRS) pH 6.1 or TRS pH 9, with or without pepsin addition (3 or 5 min).

Supplementary Table S5

Fixation (Intensity)	ICC	Agreement	Permabilization	ICC	Agreement
6-24 hours	0.58	Moderate	Proteolysis	0.71	Moderate
30-76 hours	0.64	Moderate	TRS pH 6.1<br>w/ w/o pepsin	0.77	Good
100-144 hours	0.78	Good	TRS pH 9<br>w/ w/o pepsin	0.75	Good
Global ICC	0.75	Good			

Supplementary Table S5 – Consensus. Table showing the consensus outcome of Intraclass correlation coefficients, ICC test regarding the score of interrater agreement related to Intensity. First column, fixation categories given in periods (hours) and Global ICC (overall correlation of agreement among the three raters). Second and fifth column, ICC is relative to the given fixation period or permeabilization category. Third and sixth column, agreement: interpretation of ICC. Fourth column, permeabilization categories: proteolysis, Heat-induced microRNA Retrieval using Target Retrieval Solution (TRS) pH 6.1 or TRS pH 9 with (w/) or without (w/o) pepsin.
